# Thoracic Paravertebral Mass as an Infrequent Manifestation of IgG4-Related Disease

**DOI:** 10.1155/2017/4716245

**Published:** 2017-12-28

**Authors:** Melissa Matzumura Kuan, Bernard Rubin, Alireza Meysami

**Affiliations:** ^1^Division of Rheumatology, Detroit Medical Center, Wayne State University, Henry Ford Hospital, Detroit, MI, USA; ^2^Henry Ford Medical Group, Detroit, MI, USA; ^3^Division of Rheumatology, Henry Ford Medical Group, Detroit, MI, USA

## Abstract

**Case:**

A 50-year-old African American male presented with abdominal pain and significant weight loss. On physical examination, he had parotid and submandibular gland enlargement associated with right eye proptosis. Computed tomography showed a thoracic paravertebral soft tissue mass, enlarged lymph nodes, and ascending aortic aneurysm. Laboratory results were remarkable for elevated total IgG and IgG4 subclass. The submandibular gland pathology revealed chronic sclerosing sialadenitis, with a large subset of inflammatory cells positively staining for IgG4. The histology of the paravertebral mass demonstrated fibrosclerosis with increased lymphocytic infiltrate, associated with increased IgG4 plasma cells. He was diagnosed with immunoglobulin G4-related disease (IgG4-RD). Steroid therapy initially yielded improvement; however, after steroids were stopped, there was disease recurrence. Prednisone was restarted, and the plan was to start him on rituximab. Interestingly, the patient's brother also had IgG4-RD.

**Conclusion:**

IgG4-RD can present as a paravertebral mass which is usually responsive to steroids; however, recurrent and resistant disease can be seen for which steroid-sparing agents such as rituximab should be considered. In addition, to the best of our knowledge, this is the first reported case of IgG4-RD in two family members presenting as a paravertebral mass, highlighting an exciting area for more research in the future.

## 1. Introduction

Immunoglobulin G4-related disease (IgG4-RD) is a relatively new multiorgan disease, characterized by an immune-mediated fibroinflammatory process, which can produce irreversible organ damage if untreated [1–3]. The clinical manifestations of this disease depend on the type and degree of organ involvement, making the diagnosis challenging for healthcare providers [4, 5].

Over the past few years, there has been increased understating of this condition, and the multiple disorders that were once described as isolated entities are now included under IgG4-RD spectrum [2, 3]. More importantly, there are now more therapeutic options available that can significantly change both outcome and prognosis; therefore, a correct diagnosis is even more crucial [6, 7].

We present an atypical presentation of IgG4-RD, with not only glandular and ophthalmic involvement but also thoracic paravertebral infiltration. In addition, to the best of our knowledge, this is the first reported case of IgG4-RD in two members of the same family.

## 2. Case Presentation

A 50-year-old African American male with a past medical history of hypertension, gout, sleep apnea, and hypothyroidism was evaluated for abdominal pain, associated with unintentional 20-pound weight loss, nausea, and vomiting. He denied fever, chills, night sweats, headaches, dyspnea, skin rash, arthralgias, oral ulcers, Raynaud's phenomenon, or chest pain.

On physical exam, he was found to have bilateral parotid and submandibular gland enlargement and right eye proptosis. The patient disclosed that his brother has retroperitoneal fibrosis secondary to IgG4-RD. The initial abdominal computed tomography (CT) revealed a duodenal mass. Endoscopic retrograde cholangiopancreatography (ERCP) showed a mildly dilated common bile duct, not associated with mass or stricture. A biopsy of the intra- and infra-ampullary area demonstrated active chronic duodenitis without evidence of malignancy.

**Figure 1:**
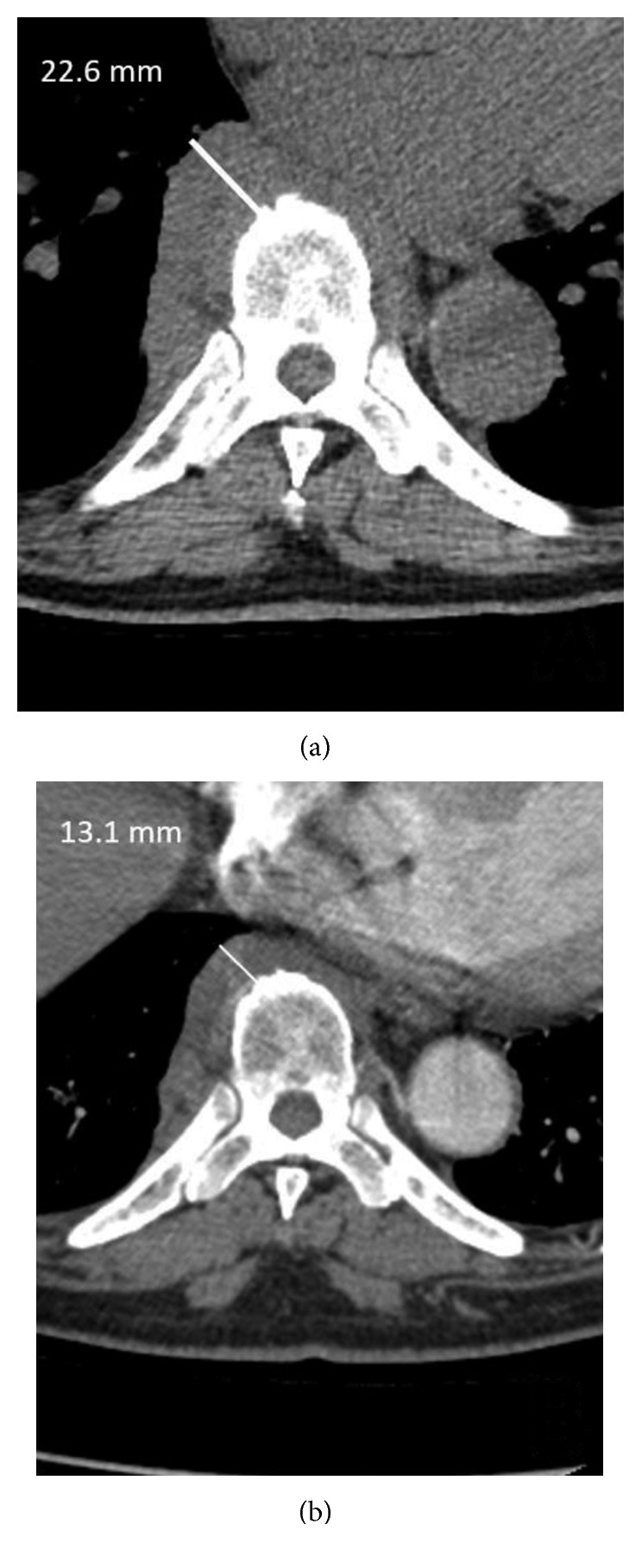
Paravertebral soft tissue mass (a) before and (b) after treatment with prednisone.

A CT of the thorax showed a confluence of elongated anterior and right paravertebral soft tissue from the midthoracic region to the diaphragmatic level, associated with mildly enlarged thoracic lymph nodes, borderline ascending aortic aneurysm, and scattered small lung nodules (Figure 1).

Complete blood count and biochemical profile were remarkable for chronic anemia and hypoalbuminemia, with normal renal and hepatic function. Monoclonal protein evaluation showed elevated IgG level of 5410 mg/dL (normal: 700–1600 mg/dL), and bone marrow biopsy demonstrated plasma cell dyscrasia (plasma cell count of 6%) with no evidence of lymphoproliferative disorder. Additional laboratory results revealed positive antinuclear antibody (ANA), low complement levels, elevated total IgG and IgG4 subclass, and elevated sedimentation rate (Table 1). The QuantiFERON^®^-TB Gold test was negative.

**Figure 2:**
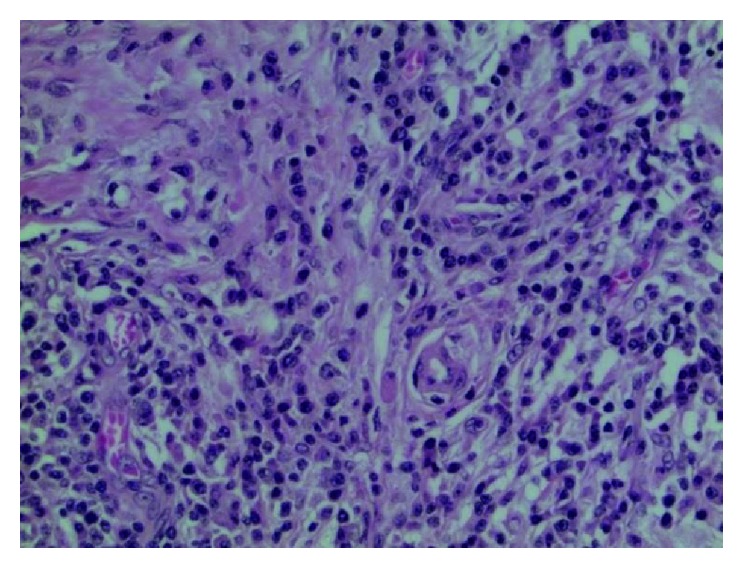
The submandibular gland shows extensive lymphoplasmacytic infiltrate intermixed with prominent fibrosis.

He underwent excision of a submandibular gland, and the pathology revealed chronic sclerosing sialadenitis, with a large subset of inflammatory cells positively staining for IgG4 (Figure 2).

**Figure 3:**
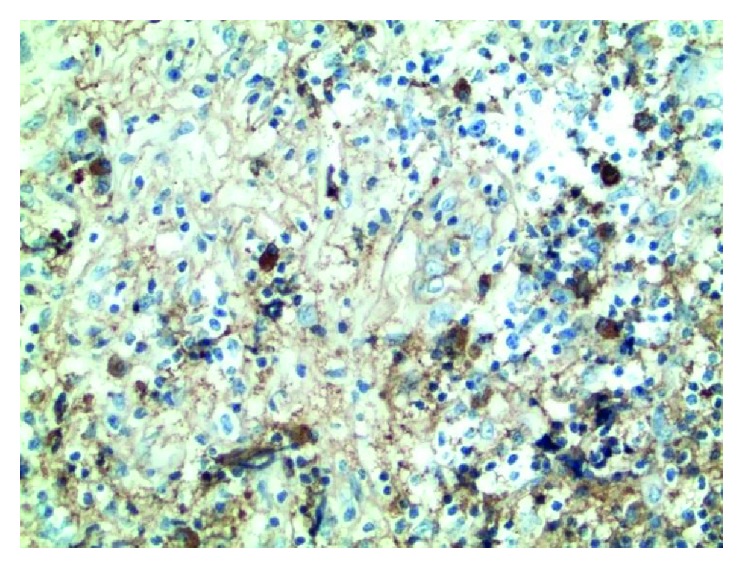
The paravertebral mass shows dense fibrosclerosis with increased lymphoplasmacytic infiltrates, and IgG4 plasma cells are slightly increased.

Subsequently, video-assisted thoracoscopy (VATS) was performed. During the procedure, a multilobar paraspinal mass near the diaphragm was noted, with pathology showing dense fibrosclerosis with increased lymphocytic infiltrates. Although staining for IgG and IgG4 was limited by the level of background staining and areas of crush artifact within the tissue, there was an increase of IgG4 plasma cells (Figure 3).

A follow-up chest CT revealed a prominent paravertebral mass. In view of this finding, the patient was started on prednisone 40 mg daily. While on prednisone, he significantly improved. A subsequent CT done 5 months later showed a decrease in the paraspinal mass (Figure 1). The serum IgG4 level also decreased significantly with prednisone treatment (Table 2). Unfortunately, after prednisone was discontinued, the IgG4 level increased, and there was slight progression in thickness of the paravertebral soft tissue.

Prednisone was restarted, and rituximab therapy was planned.

## 3. Discussion

IgG4-RD is a relatively new systemic fibroinflammatory disease. It is more common in male patients above age 50 [1, 2]. The pathophysiology is poorly understood, but genetic, infectious, and autoimmune factors may play a role [3]. The most common manifestations are type 1 autoimmune pancreatitis, sialadenitis, dacryoadenitis, tubulointerstitial nephritis, retroperitoneal fibrosis, and lymphadenopathy [2, 4, 6].

The diagnosis is based on clinic-pathologic correlation. Clinical manifestations depend on the type and degree of organ involvement. Solid organ involvement commonly presents as an inflammatory pseudotumor [8, 9]. Laboratory findings are nonspecific. High serum IgG4 level defined as IgG4 ≥ 135 mg/dL is present in most of the cases [3]. Acute reactive markers can be highly elevated or normal, and complement levels can be low.

The gold standard for diagnosis is biopsy, which also can differentiate this entity from other conditions such as lymphoma [1, 3]. The typical pathological findings are (1) diffuse lymphoplasmocytic infiltrates rich in IgG4 plasma cells (more than 10/high power field) and/or tissue IgG4/IgG ratio more than 40%, (2) storiform fibrosis, and (3) obliterative phlebitis.

Our patient presented with a paravertebral mass as a manifestation of IgG4-RD. There are only a few case reports that describe this atypical presentation [1, 4, 9, 10]. Watanabe et al. presented a unique familiar case report of type 1 autoimmune pancreatitis associated with IgG4-related sialadenitis and retroperitoneal fibrosis [11]. Similarly, our patient's brother has symptoms suggestive of IgG4-RD, and if true, this would represent another example of the possible genetic link in this illness.

Early recognition and treatment can prevent permanent organ damage since chronic inflammation leads to a tumefactive mass that can destroy the involved organ [1, 3]. There are no randomized controlled trials for IgG4-RD treatment. The International Consensus Guidance statement recommends initiation of treatment in all symptomatic patients, and glucocorticoids are the first-line agent for remission induction. However, patients can relapse after prednisone is tapered or discontinued. The use of steroid-sparing agents is controversial, and rituximab is an option for refractory cases [1, 5, 6]. The study conducted by Carruthers et al. showed 97% complete remission after two doses of rituximab [7], using serum IgG4 level as an important tool to assess treatment response and to predict possible relapse [12].

In conclusion, systemic involvement and possible permanent organ damage from IgG4-RD can present insidiously, so all physicians should be aware of this infrequent but important condition since early diagnosis and treatment may alter the prognosis of this multisystem disease. In addition, if there is a family history of IgG4-RD, this should not deter one from considering the diagnosis as our interesting case illustrates.

## Figures and Tables

**Table 1:** Baseline autoimmune workup.

ANA	1 : 640
c-ANCA	<1 : 20
p-ANCA	<1 : 20
Rheumatoid factor (IU/mL)	<15
Smith antibody (units)	3
RNP antibody (units)	4
SSA/Ro antibody (units)	10
SSB/La antibody (units)	4
C3 (mg/dL)	40
C4 (mg/dL)	6
Sedimentation rate (mm/Hr)	72

**Table 2:** Laboratory results before and after prednisone (prednisone was started on June 2015 and discontinued on November 2016).

	April 2014	June 2015	December 2015	February 2016	April 2016	June 2016	September 2016	December 2016
IgG4 (mg/dL)	4850	494	531	397	462	537	760	1210
Sedimentation rate	28	72	26	11	—	18	—	41
CRP	<0.3	<0.3	<0.3	<0.3	0.1	<0.1	—	0.1
